# Hyperemesis gravidarum and oral health: A scoping review

**DOI:** 10.1002/ijgo.70958

**Published:** 2026-03-31

**Authors:** Niamh Coffey, Lucy James, Angela C. Flynn, Zeinab El‐Dirani, Shahnawaz Khijmatgar

**Affiliations:** ^1^ School of Dentistry Royal College of Surgeons in Ireland Dublin Ireland; ^2^ School of Medicine Royal College of Surgeons in Ireland Dublin Ireland; ^3^ School of Population Health Royal College of Surgeons in Ireland Dublin Ireland

**Keywords:** dental erosion, hyperemesis gravidarum, oral health, pregnancy complications

## Abstract

**Background:**

Hyperemesis gravidarum (HG) is a severe form of nausea and vomiting in pregnancy (NVP), associated with dehydration, nutritional deficiencies, and systemic complications. Although recurrent vomiting, altered dietary patterns, and medication effects might plausibly affect oral health, this relationship has not been comprehensively synthesized.

**Objective:**

The objective of this scoping review is to systematically map the existing evidence on the association between HG and oral health.

**Eligibility Criteria:**

Primary studies of any design examining oral health in individuals with HG or severe nausea and vomiting in pregnancy (meeting HG‐level severity criteria) were included. Editorials, commentaries, studies of mild or moderate nausea and vomiting in pregnancy (NVP), and studies without oral health findings were excluded.

**Sources of Evidence:**

Seven electronic databases (PubMed/MEDLINE, Embase, CINAHL, Scopus, Web of Science, OVID Medline, and the Cochrane Library) were searched in October 2025. Additional sources included gray literature from HG‐focused organizations (e.g., HER Foundation and Pregnancy Sickness Support) and reference lists of included studies.

**Charting Methods:**

Screening was conducted independently by two reviewers following reviewer calibration, using Covidence software for reference management and deduplication. Full texts were assessed independently by two reviewers, with disagreements resolved through discussion or third‐reviewer adjudication. A pre‐piloted data charting form was used to extract study characteristics, oral health assessment methods, and key findings related to HG. Data were synthesized narratively and reported in accordance with Preferred Reporting Items for Systematic Reviews and Meta‐Analyses extension for Scoping Reviews guidelines.

**Results:**

The search identified 2697 records, with 1752 remaining after deduplication. Of 38 full‐text articles screened, 10 studies met the inclusion criteria. Study designs included cross‐sectional (*n =* 3), case reports (*n =* 3), case–control (*n =* 2), and retrospective cohort studies (*n =* 2). Across all studies, a total of 8630 participants were represented (range 1–7765; median 34.5). Periodontal status was clinically assessed in one study, which found significantly worse periodontal indices in HG compared with controls, and one additional study reported higher self‐reported gum disease in individuals with HG. Dental cavities were self‐reported in one study, showing higher pre‐ and post‐pregnancy prevalence among those with HG. Three studies described hypersalivation consistent with ptyalism gravidarum, frequently co‐occurring with HG, while salivary biomarkers were assessed in two studies. One study reported enamel hypoplasia in children born to mothers with severe HG. Self‐reported oral hygiene difficulties were described in six studies, and one study noted higher levels of self‐reported temporomandibular joint disorder in HG participants.

**Conclusion:**

This scoping review identifies a substantial gap in evidence linking HG with oral health conditions. Available studies suggest challenges in maintaining oral hygiene practices, alongside frequent gastric acid exposure, which might heighten risk for oral complications, but robust clinical data are lacking. Further research and interdisciplinary attention are needed to clarify and address these associations.

## 1 BACKGROUND

Hyperemesis gravidarum (HG) is a debilitating condition characterized by severe nausea and/or vomiting that affects quality of life. It is defined by onset in early pregnancy, specifically before a gestational age of 16 weeks, involves severe nausea and/or vomiting and results in the inability to eat and/or drink normally.^1^ The prevalence of HG is estimated to be between 0.3% and 10.8% of pregnancies.^2^ Generally, HG causes significant weight loss, dehydration, electrolyte imbalances, and nutritional deficiencies. HG significantly affects maternal physical and psychological well‐being.^3^ Although the effects of HG have been reported, including its impact on nutrition, hydration, and mental health, there is a significant gap in evidence specifically related to the association between HG and oral health.

Clinical guidelines highlight that persistent vomiting, as seen in conditions like HG, can result in dental erosion due to exposure to gastric acid.^4^ The frequency and severity of dental erosion might not always correlate linearly with the number of vomiting episodes, suggesting variability in individual susceptibility and additional contributing factors.^5^ Thus, while HG affects maternal general health, it might also have secondary effects on dental/oral health, primarily through acid‐induced erosion.

Although case reports^6^ and anecdotal observations have suggested that individuals with HG might encounter significant difficulties in maintaining oral hygiene and might develop distinctive oral complications, these reports remain inconsistent and have not been systematically synthesized. In contrast, the broader literature on pregnancy and oral health is relatively well established. Hormonal fluctuations during pregnancy might lead to recognized conditions such as pregnancy epulis, while chronic acid exposure in other contexts, for example, gastroesophageal reflux disease and bulimia nervosa, is strongly associated with dental erosion.^7^, ^8^ Similarly, dietary modifications, including increased intake of fermentable carbohydrates, are well known to exacerbate caries risk.^9^ Given the defining pathophysiological features of HG, namely recurrent and often severe vomiting,^1^ altered nutritional intake,^10^ and pharmacological interventions that can impair salivary flow (e.g., promethazine), it is reasonable to hypothesize that HG constitutes a distinct risk profile for adverse oral health conditions. However, the relationship between HG and oral health has not been systematically examined, and existing studies have not established whether any associations are unidirectional or bidirectional.

Therefore, the objective of this scoping review was to systematically map the existing evidence on the association between HG and oral health measures.

### 1.1 Research question

What evidence is available on the association between HG and oral health conditions?

## 2 METHODS

### 2.1 Protocol registration

The full protocol is drafted as per the Preferred Reporting Items for Systematic Reviews and Meta‐Analyses (PRISMA) checklist for conducting scoping reviews^11^; refer to Appendix S1. The protocol was registered on Open Science Framework (OSF) (10.17605/OSF.IO/8HNP5).

### 2.2 Eligibility criteria

Studies were included if they addressed at least one oral health areas for women who experienced severe NVP (SNVP) or HG. All types of study designs, with no restriction to quality or setting, were eligible. The review was limited to articles in English. The review question was structured using the PCC framework as follows:

Population (P): Pregnant individuals with HG or SNVP.

Concept (C): Oral health and oral hygiene.

Specific dental conditions:

- dental erosion
- dental caries
- periodontal disease.

Salivary gland function (e.g., xerostomia, hypersalivation).

Context (C): Any study design investigating the association, including:

- observational studies
- case reports/case series
- qualitative studies
- reviews.

### 2.3 Inclusion criteria

Studies were included if they:

- Involved human participants with HG, severe NVP, or other terms indicating severe pregnancy sickness.
- Provided evidence of severity through:
  ○ clinical markers (e.g., significant weight loss, dehydration, electrolyte imbalance, hospitalisation, intravenous fluids, pharmacological therapy), or
  ○ severe functional impact (e.g., inability to eat or drink, significant disruption to daily life, inability to perform usual activities)
- Described symptom onset before 16 weeks' gestation
- Were peer‐reviewed journal articles, written in English.
- Reported or measured any oral health condition, including but not limited to:
  ○ dental caries
  ○ dental erosion
  ○ periodontal diseases
  ○ alterations in salivary function
  ○ documented challenges with oral hygiene practices
- Included quantitative, qualitative, mixed‐methods studies, case reports and case series.

### 2.4 Exclusion criteria

Studies were excluded if they:

- focused solely on general pregnancy‐related oral health
- investigated only mild or moderate NVP that does not meet the HG definition
- addressed systemic effects of HG without specific mention of oral health conditions
- were non‐human studies.
- were opinion pieces without supporting data.

### 2.5 Search strategy

#### 2.5.1 Information sources

The following databases were searched: PubMed/MEDLINE, Embase, CINAHL, Scopus, Web of Science, OVID Medline, and the Cochrane Library. Additional sources included the reference lists of included studies and relevant reviews. A gray literature search was conducted via websites of key organizations dedicated to HG research and support (e.g., HER Foundation and Pregnancy Sickness Support).

### 2.6 Search strategy

Search strategies were developed collaboratively by two authors using a combination of MeSH terms and free‐text keywords for *hyperemesis gravidarum* and *oral health*. The strategies were refined through pilot searches and team discussion, and the final versions for each database were documented for transparency. The search was conducted in October 2025 and search results were exported into Covidence systematic review software (Veritas Health Innovation, Melbourne, Australia), which was used to manage references and remove duplicates. Full search strategies are provided in Appendix S2.

### 2.7 Selection of sources of evidence

To ensure consistency, reviewers (N.C., S.K., and L.J.) initially screened a calibration set of 20 randomly selected records, followed by group discussion to refine the screening manual. Titles and abstracts were then screened by two reviewers, with any study marked as potentially relevant by at least one reviewer advancing to full‐text review. Full texts were also assessed by two reviewers against the eligibility criteria. Discrepancies were resolved through discussion or, if required, adjudication by a third reviewer. Reasons for exclusion at the full‐text stage were recorded in detail.

### 2.8 Data extraction

A pre‐piloted data charting form was used to systematically extract information from each study. The extraction of the data was performed by two authors (S.K. and N.C.). Extracted items included study identification details (author[s], year of publication, journal, and country of origin), study characteristics (design, aims, and population), and the oral health conditions investigated (e.g., dental caries, erosion, and salivary changes) together with the methods used to assess them. Key findings directly linking HG to oral health, including any reported statistical significance, were recorded, along with any mechanisms or explanatory factors proposed by the original authors.

### 2.9 Synthesis of results

Extracted data were synthesised descriptively and organized according to study design, oral health conditions, and assessment methods. A narrative synthesis approach was used to summarize patterns, map the range of reported oral health effects, and compare findings across studies. Due to the heterogeneity of study designs, populations, and outcome measures, no quantitative synthesis or meta‐analysis was undertaken.

This scoping review was conducted and reported in accordance with the PRISMA‐ScR (Preferred Reporting Items for Systematic Reviews and Meta‐Analyses extension for Scoping Reviews) guidelines (Appendix S1).

## 3 RESULTS

### 3.1 Study types

The results of the search yielded 2697 articles, with 1752 remaining following deduplication. Following title and abstract screening, the full texts of 38 articles were screened, and a total of ten studies met the inclusion criteria and were included in this scoping review,^12^, ^13^, ^14^, ^15^, ^16^, ^17^, ^18^, ^19^, ^20^, ^21^ Figure 1.

**FIGURE 1:**
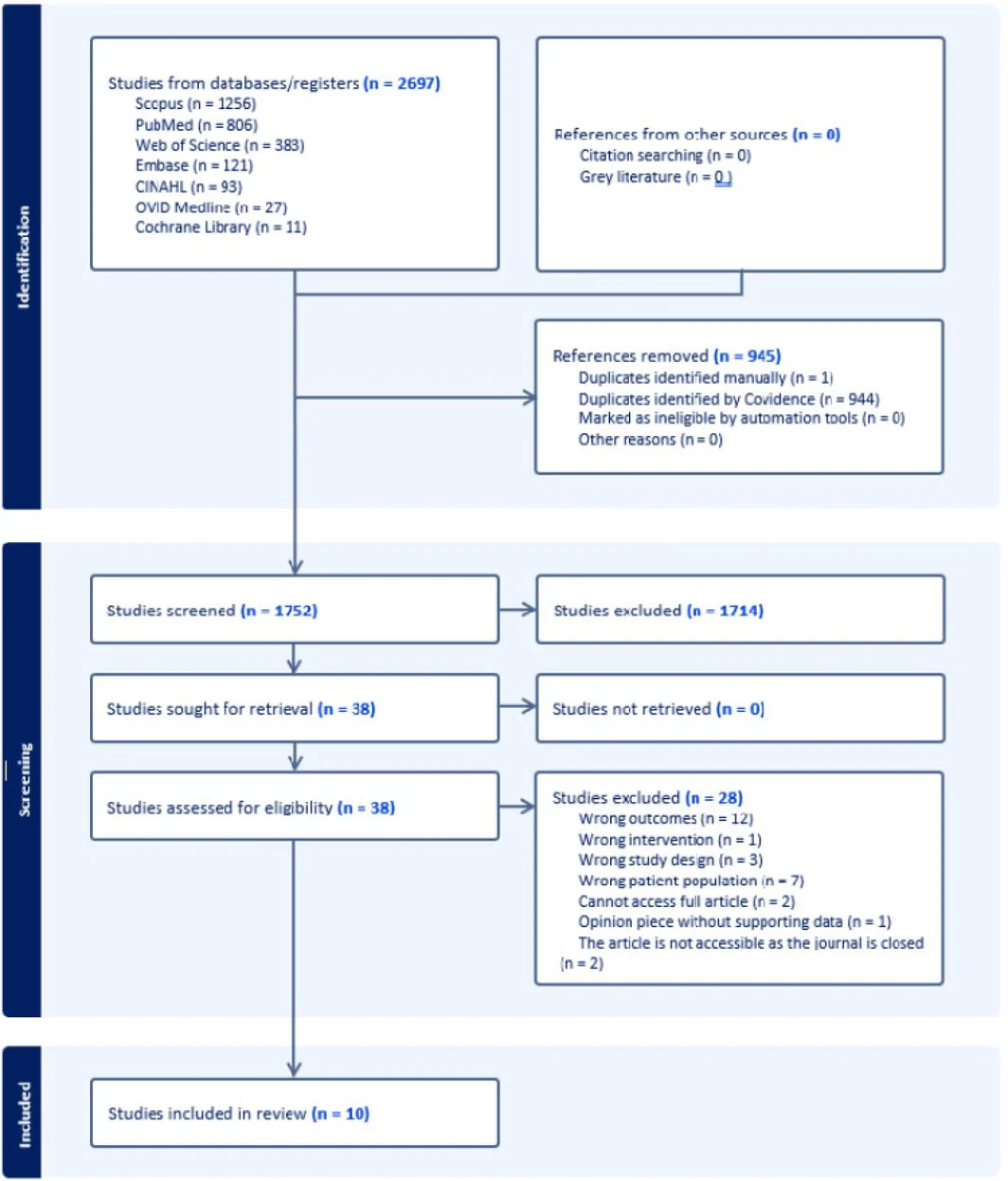
PRISMA flow diagram of study selection.

### 3.2 Characteristics of included studies

The studies were published between 1990 and 2021 and were conducted in Turkey, Japan, Israel, the USA, Malaysia, and the UK. Among the studies, 30% (*N =* 3) were cross‐sectional studies, 30% (*N =* 3) were case‐reports, 20% (*N =* 2) were case–control studies, and 20% (*N =* 2) were retrospective cohort studies. Across 10 studies, a total sample size of 8630 participants was observed, with a mean sample size of 863 participants per study (with studies ranging from one participant and the largest encompassing 7765 participants). These samples vary among different study designs (Appendix S3).

The samples included in the studies had a mean age of 28.72 ± 2.47 years with a median of 28 years (range 23.5–32 years). HG patients tended to be slightly younger but no significant age differences existed between HG and controls.

The relationship between HG and oral health conditions was reported in all included 1 studies. Oral health conditions assessed across studies included periodontal indices, dental caries, enamel defects in children, salivary biomarkers, hypersalivation/ptyalism gravidarum (PG), self‐reported oral hygiene difficulties, temporomandibular joint symptoms, and isolated oral pathology findings. Details of individual study characteristics are presented in Appendix S1 and S2.

### 3.3 Oral conditions

Periodontal status was formally assessed in only one study.^13^ Altaş (2020) performed a standardized clinical periodontal examination and found that women with HG had significantly higher Plaque Index and Gingival Index scores, increased Bleeding‐on‐Probing, and a greater number of sites with probing depth ≥ 4 mm compared with controls, indicating worse periodontal health (gingivitis and periodontitis) in the HG group. Another study reported higher self‐reported levels of gum disease in the post‐pregnancy HG group (*P* = 0.0075; OR = 3.52).

Dental caries were examined in one study.^15^ Tian (2017) used self‐reported dental history and found a higher pre‐pregnancy prevalence of dental cavities in women with a history of HG than in controls (36.3% vs. 28.8%; OR = 1.41; *P* = 0.015). This was one of the seven most commonly reported conditions post‐HG pregnancy. No study performed clinical caries examinations or radiographic assessments, so caries progression or severity could not be determined.

One study^21^ evaluated enamel hypoplasia in offspring via visual and photographic examination and reported a higher frequency of enamel defects among children born to mothers who experienced severe HG. Maternal dentition was not examined in this study.

Self‐reported temporomandibular joint disorder (TMJD) levels were higher in the HG participants compared to controls (10% HG, 2.4% non‐HG, *P* < 0.0001, OR 4.54).^15^

### 3.4 Salivary symptoms

Hypersalivation consistent with PG was reported in three studies.^14^, ^17^, ^18^ In all three, PG was characterized by persistent excessive salivation requiring frequent expectoration and the use of spit cups, tissues, or cloths, typically beginning in early pregnancy and, in many cases, continuing until delivery. Reported salivary volume was patient estimated (e.g., 1–1.5 L/day) in Suzuki^18^, while another study^14^ referenced volumes ≥2 L/day from existing literature; however, no study objectively measured salivary flow. PG frequently co‐occurred with HG, with one study reporting a strong association (^17^; adjusted OR 13.5, *P* < 0.01) and another identifying coexistence in approximately 40% of PG cases.^14^ Functional effects reported across studies included disrupted sleep, difficulty swallowing saliva, and challenges maintaining oral hygiene, although no dental or periodontal indices were assessed.

Another study^19^ reported marked sialorrhea (estimated at approximately 1.5–2 L/day) and, conversely, episodes of xerostomia associated with HG treatment but did not classify cases as PG. Across all studies describing salivary symptoms, severity assessments were based on self‐report or clinical observation, and no study evaluated salivary composition, buffering capacity, or associated changes in oral tissues.

### 3.5 Self‐reported oral hygiene difficulties

Self‐reported oral hygiene difficulties were noted in six of the 10 studies. One study^13^ reported insufficient tooth‐brushing due to gagging and vomiting, although no formal questionnaire was used. Another study^14^ described social distress, the need for cups or tissues, and sleep disruption due to excessive salivation. Beevi (2015) noted constant spitting causing embarrassment and challenges in work and social settings. Suzuki's studies in 2009 and 2013 reported the need for spitting cups, tissues, or cloths throughout pregnancy, along with a bad taste and difficulty swallowing thickened saliva. Freeman 1994 (24) documented a bitter taste, frequent expectoration, and excoriation linked to oral hygiene challenges.

### 3.6 Oral pathology

Two studies made a comment about other oral pathologies.^16^ ^,^ ^20^ One study^16^ ruled out oral infection through clinical examination and laboratory tests, indicating no significant findings. Another study^20^ reported excoriations in the buccal cavity and angles of the mouth in one case, suggesting localized oral tissue damage potentially related to HG.

### 3.7 Oral health assessment methods

Oral health were assessed using different methods in the included 10 studies, with five studies using clinical examinations and self‐reported data. Only one study^13^ used a standardized clinical periodontal examination by a single blinded examiner at six sites per tooth with a Williams probe, measuring Plaque Index, Gingival Index, Probing Pocket Depth, and Bleeding‐on‐Probing Index to assess gingivitis and periodontitis.

Other studies relied on clinical observations and patient self‐reports.^13^, ^16^, ^18^, ^19^ Tian (2017) combined clinical examinations with self‐reported symptoms via an online survey (60 pre‐ and 80 post‐pregnancy items). Beevi 2015 used retrospective chart analysis to evaluate oral health. Köşüş et al.^11^ analyzed fasting whole‐saliva samples for gustin (CA‐6) levels using a commercial ELISA kit. Suzuki (2009) measured serum total and pancreatic amylase, focusing on systemic biomarkers. Nozaka (1990) assessed enamel hypoplasia in children via clinical visual inspection, photographic evaluation, and questionnaires, linked to maternal HG.

### 3.8 Synthesis of results

Across the included studies, HG was consistently associated with a range of oral health challenges, including worsened periodontal indicators, increased self‐reporting of dental cavities and TMJD, frequent reports of hypersalivation or ptyalism, self‐reported oral hygiene difficulties, and enamel defects in offspring. However, the heterogeneity of study designs, inconsistent diagnostic criteria, and reliance on self‐reported measures limited direct comparability. Collectively, the findings suggest potential pathways linking HG to oral complications, primarily through acid exposure, altered salivary function, and disrupted oral hygiene, although robust clinical evidence remains sparse (noting that this is based on a descriptive and exploratory assessment of the literature; a formal risk‐of‐bias assessment was not carried out).

## 4 DISCUSSION

This scoping review identified 10 studies investigating the relationship between HG and oral health conditions. Overall, the available evidence was limited, methodologically heterogeneous, and largely descriptive. As this was a scoping review, no formal risk‐of‐bias assessment was undertaken; therefore, observations regarding the strength or limitations of the evidence are exploratory and based on study design and reporting rather than on formal quality grading.

Within the included studies, only one study used standardized clinical periodontal indices, demonstrating poorer periodontal parameters in women with HG compared with controls. A single survey‐based study reported a higher prevalence of self‐reported dental caries among women with HG both before and after pregnancy, although this was not clinically verified.^15^ No study examined dental erosion, despite the recognized potential for gastric acid exposure in HG to cause enamel loss. One older observational study suggested an association between severe maternal HG and enamel hypoplasia in offspring, but maternal oral status was not evaluated.^21^

Across multiple studies, women with HG frequently described difficulties maintaining oral hygiene, often related to gagging, nausea, and hypersalivation. PG and sialorrhea were common complaints, sometimes co‐occurring with HG, but no study objectively measured salivary flow or composition. The findings indicate that HG is associated with multiple self‐reported functional and behavioral oral challenges, but direct evidence of oral disease burden remains scarce, indicating a lack of research into this area.

The findings highlight several biologically plausible mechanisms through which HG could adversely affect oral health, although the extent of resulting clinical disease has not yet been well quantified. Chronic and severe vomiting exposes the oral cavity to gastric acid, creating conditions conducive to dental erosion, dentin sensitivity, and restorative material degradation. Although none of the included studies assessed erosion directly, evidence from other acid‐mediated dental conditions suggests that repeated acidic exposure during early pregnancy could have measurable effects on enamel over time.^8^, ^22^ This represents a major gap in the HG‐specific evidence base.

Functional and behavioral constraints also appear central. Women with HG frequently reported reduced toothbrushing, gagging during oral hygiene, and avoidance of toothpaste flavors, contributing to plaque accumulation and periodontal inflammation. Of concern is that this sensory sensitivity to toothbrushing sometimes extends postnatally.^23^ The significantly poorer periodontal indices identified in the only study using standardized measurements (Altaş 2020) can be expected with these behavioral patterns.

Salivary alterations are another likely mechanism. Reports of PG, sialorrhea, and, conversely, treatment‐related xerostomia suggest substantial disruption to normal saliva handling and composition. Saliva plays a critical protective role in buffering acid, clearing debris, and maintaining enamel integrity; therefore, alterations in its volume, viscosity, or pH might worsen susceptibility to dental pathology.^24^ Yet no study measured salivary pH or buffering capacity, leaving a key physiological gap.

Two additional case reports were identified but could not be fully extracted for detailed data analysis. These reports describe acute bilateral temporomandibular joint dislocation during forceful emesis and mucosal hemorrhage secondary to acquired vitamin K deficiency in severe HG.^25^, ^26^ Although not included in the main evidence mapping, they highlight the potential for rare but serious oral and maxillofacial complications in extreme cases.

From a clinical perspective, the scoping review findings suggest the need for integrated antenatal care pathways. Dental practitioners should be aware that women with HG might struggle to maintain routine oral hygiene and might require modified preventive strategies, such as non‐foaming toothpastes, high‐fluoride formulations, neutralizing mouth rinses, or tailored dietary counseling.

Preventive recommendations, however, are largely extrapolated from broader guidance for chronic vomiting and erosive tooth wear rather than from HG‐specific interventional studies. Current preventive guidance^27^ for individuals with chronic vomiting, including vomiting in pregnancy, recommends avoiding toothbrushing immediately after vomiting and rinsing with water to minimize abrasion of acid‐softened enamel. Where tolerated, applying fluoride toothpaste with a finger or using a fluoride mouth rinse can provide topical protection when brushing is not feasible. Where feasible, professional fluoride varnish application could be considered to enhance remineralization during periods of frequent vomiting, although its effectiveness has not been specifically evaluated in HG populations. Obstetric teams should consider early dental referral for women with severe or prolonged HG. Interdisciplinary antenatal oral health screening might be beneficial, particularly in high‐risk pregnancies involving recurrent vomiting.

### 4.1 Strengths and weaknesses of the study

To our knowledge, this review provides the first synthesis focused specifically on the intersection between HG and oral health. By including diverse study designs, including case reports, cross‐sectional, case–control, and cohort studies, it captures the full extent of published data and highlights consistent symptom patterns across different populations and decades. The review highlights how limited the existing research is and draws attention to a clinically relevant but underexplored area.

There were some limitations, mainly due to the lack of research in this area. First, only English language studies were included. Only one study used standardized dental indices, and the majority relied on self‐reported oral symptoms, limiting the strength of causal inference. Finally, none of the studies examined long‐term oral health conditions beyond pregnancy.

### 4.2 Future research

Future research should prioritize robust clinical assessment of dental erosion, salivary function, periodontal conditions, and the effectiveness of preventive interventions, as these areas remain largely unquantified in the existing literature.

## 5 CONCLUSION

This scoping review highlights a substantial gap in the evidence linking HG with oral health conditions. The limited available studies suggest that individuals with HG often experience significant disruption to oral hygiene practices and frequent exposure to gastric acid, both of which might have implications for dental and oral soft‐tissue complications. However, robust clinical data quantifying these conditions are lacking. Future research should prioritize well‐designed studies that evaluate oral disease prevalence, salivary changes, and erosion risk in HG. Greater attention to oral health assessment and tailored preventive advice during antenatal care might be warranted for individuals experiencing severe nausea and vomiting in pregnancy.

## AUTHOR CONTRIBUTIONS

N.C. contributed to the conceptualization, methodology, protocol registration, literature searching, screening, data curation, and led the writing of the manuscript from original draft through final revision. S.K. contributed to protocol registration, literature searching, screening, and assisted in drafting and revising the manuscript. L.J. contributed to the literature search and screening and participated in manuscript review and editing. Z.E.‐D. and A.C.F. contributed to methodology and protocol development and provided critical review and editing of the manuscript. All authors approved the final manuscript. Covidence was used for screening management. All decisions and synthesis were conducted by the authors. No generative AI tools were used.

## FUNDING INFORMATION

One author (ZED) is supported by a SPHeRE program scholarship (Health Research Board, Ireland, under grant SPHERE/2022/001). No additional external funding was received for this study.

## CONFLICT OF INTEREST STATEMENT

The authors declare that they have no conflicts of interest, financial or otherwise, related to this work. All authors approved the final manuscript.

## Supporting information


Appendix S1.

Appendix S2.

Appendix S3.

## Data Availability

Data sharing is not applicable to this article as no new data were created or analyzed in this study.
