# Supplementary material for: Hyperemesis gravidarum and oral health: A scoping review
Source: Int J Gynaecol Obstet. 2026 Mar 31;175(1):48–55. doi: 10.1002/ijgo.70958 (PMC13629587; doi:10.1002/ijgo.70958)
Supplement: Supplementary file 1 [file IJGO-175-48-s001.docx]

Appendix 3: Characteristics of Included Studies

| **Study ID** | **Author(s)** | **Country** | **Journal** | **Year** | **Study design** | **Sample size** | **Age** | **Gestational age at diagnosis** | **Diagnosis of HG criteria** |
| --- | --- | --- | --- | --- | --- | --- | --- | --- | --- |
| 1 | Köşüş ,2021 | Turkey | Annals of Clinical and Analytical Medicine | 2021 | Prospective case-control study | 60 women initially (30 HG, 30 controls); final analysis on 24 HG and 16 controls after dropouts | Mean=29 yrs; no significant difference between groups | 6–14 weeks | Persistent nausea + vomiting plus ketosis plus ≥ 5 % pre-pregnancy weight loss |
| 2 | Altas,2020 | Turkey | European Journal of General Dentistry | 2020 | Cross-sectional comparative study | 60 pregnant women (30 HG, 30 healthy pregnant) | Mean 29 ± 3 yr (HG) vs 28 ± 4 yr (controls); range 18–40 yr | 8th–10th week of pregnancy | Presence of ALL: ≥1+ ketonuria, > 5 % weight loss, and vomiting ≥ 2×/day |
| 3 | Bronshtein,2018 | Israel (single private clinic, Haifa) | Israel Medical Association Journal (IMAJ) | 2018 | Retrospective cohort | 22 women with ptyalism gravidarum (PG) (49 pregnancies); 1 lost to follow-up | 67 % aged 25–35 yr | Symptom onset: 4–6 week (37 %), 8 week (55 %), 13 week (8 %) | Vomiting-induced weight loss ± ketonuria (applied retrospectively via chart/phone) |
| 4 | Tian,2017 | USA | Autonomic Neuroscience: Basic and Clinical | 2017 | Case-control survey study | 449 women with HG (IV fluids / TPN / NG tube) | HG group younger, heavier, more often white; mean first-pregnancy year 2003 vs 2002 controls | Symptoms began in 1st trimester and continued until 35–36 week (Case 1) or delivery (Case 2) | Severe nausea/vomiting + IV fluids or TPN/NG feeding |
| 5 | Beevi,2015 | Malaysia | American Journal of Clinical Hypnosis | 2015 | Case report | 1 | 28 years | Ptyalism at 8 weeks; Hyperemesis at 12 weeks | Severe nausea and vomiting after every meal, weight loss, poor diet |
| 6 | Suzuki,2013 | Japan | Archives of Gynecology and Obstetrics | 2013 | Retrospective cohort study | 22 cases with ptyalism gravidarum; 7,743 controls | Mean age: 31 years (cases), 32 years (controls) | Symptoms continued until delivery (identified from early pregnancy) | Severe nausea, vomiting, >5% weight loss requiring IV fluids/nutrition |
| 7 | Suzuki,2009 | Japan | North American Journal of Medical Sciences | 2009 | Case report (n = 2) | 2 pregnant women with ptyalism gravidarum (PG) persisting beyond 1st trimester | Case 1: 34yrs; Case 2: 23 yrs | Symptoms began in 1st trimester and continued until 35–36 week (Case 1) or delivery (Case 2) | Severe nausea + vomiting + excessive salivation (1–1.5 L/day) |
| 8 | Robertson,1999 | Scotland, UK | International Journal of Eating Disorders | 1999 | Cross-sectional comparative study (three parallel groups) | 29 hyperemesis gravidarum (HG) patients | HG 29yrs; Bulimics 23–24; Controls 28 | HG 10.5 wks; Controls 7.6 wks | Clinical admission for repeated vomiting in pregnancy; no binge eating, alcohol excess or pre-existing illness |
| 9 | Freeman,1994 | USA (New York), patients of Haitian origin | Journal of the National Medical Association | 1994 | Case reports (2 patients) | 2 | 30 years (Case 1), 31 years (Case 2) | First trimester (both cases) | Severe nausea, vomiting, weight loss, excessive salivation (1.5–2 L/day) |
| 10 | Nozaka,1990 | Japan | The Japanese Journal of Pediatric Dentistry | 1990 | Nationwide field survey (observational, cross-sectional) | 141 children with enamel hypoplasia; 120 controls | ≥3 years (primary dentition) | Not directly applicable; enamel hypoplasia linked to fetal, neonatal, and infantile stages | Not directly assessed; maternal history included severe hyperemesis gravidarum |

|  | **Intervention/exposure (If any specific treatment for HG or relevant exposure)** | **Dental Indices used for oral health outcomes** | **Dental Caries** | **Dental Erosion** | **Gingivitis** | **Periodontitis** | **Xerostomia** | **Hypersalivation** | **Oral infections** | **Self-reported oral hygiene difficulties** | **Measurement method of oral health outcomes (Clinical examination, self-reported questionnaire, salivary flow measurement, dental indices used)** | **What the study explicitly reported regarding the relationship between HG and the specific oral health outcome(s).** | **Statistical Significance/Effect Size (if reported)** | **Nature of the Relationship: (e.g., positive association, no association, protective effect).** |
| --- | --- | --- | --- | --- | --- | --- | --- | --- | --- | --- | --- | --- | --- | --- |
| Köşüş, 2021 | Patient with HG-Exposure | None-study focused on salivary gustin (CA-6) levels | Not reported | Not reported | Not reported | Not reported | Not reported | Not reported | Not reported | Not reported | Fasting whole-saliva samples collected in the morning, centrifuged, and analysed for gustin (CA-6) by commercial ELISA kit | Salivary gustin (CA-6) was lower in HG women (1.38 ng/mL) than controls (2.14 ng/mL), but the difference was not statistically significant (p = 0.149). | Mean difference: 0.76 ng/mL ↓ in HG (ns) | No association-gustin levels did not significantly differ between groups. |
| Altas,2020 | H/O of HG | Plaque Index (PI), Gingival Index (GI), Probing Pocket Depth (PPD), Bleeding-on-Probing Index (BOPI) | Not reported | Not reported | Reported via GI & BOPI; gingivitis defined as bleeding on > 10 % of surfaces with no periodontal pockets | Defined as ≥ 2 sites with PPD ≥ 4 mm | Not reported | Not reported | Periodontal disease examined; other oral infections not reported | Authors note “insufficient tooth-brushing” due to gagging/vomiting, but no formal questionnaire used | Standardized clinical periodontal examination by a single blinded examiner at six sites per tooth using Williams probe | “PI, GI, PPD, and BOPI were significantly higher in HG group compared to controls.” Prevalence of periodontitis: 63.3 % HG vs 20 % controls. | P-values: PI = 0.04, GI = 0.009, PPD < 0.001, BOPI = 0.03; periodontitis prevalence χ² P < 0.001 | Positive association-HG group showed worse periodontal status than controls. |
| Bronshtein,  2018 | No standardised intervention. Two women reported complete symptom resolution after hypnosis + acupuncture. Other supportive measures noted. | No dental indices. study focuses on PG (hypersalivation); no periodontal or caries indices. | Not reported | Not reported | Not reported | Not reported | Not reported | Primary outcome-defined as excessive salivation requiring expectoration; volumes ≥ 2 L/day described in literature. | Not reported | Not formally assessed, but women described social distress, need for cups/tissues, sleep disruption. | No dental indices; diagnosis and severity based on patient report and clinical observation (carrying spit cup/tissues). | 40 % of PG cases (11/22) also had HG. PG prevalence among women with HG in cohort = 2.3 % vs 0.09 % in women without HG. No direct oral health indices measured, but coexistence of PG and HG documented. | higher rate of HG in primary PG group (69 % vs 14 % secondary PG). | Positive association- PG frequently coexists with HG, but study does not assess impact on oral tissues. |
| Tian,2017 | Exposure=HG Diagnosis | None | Pre-pregnancy: 36.3 % vs 28.8 % controls (OR 1.41; p = 0.015) reported | Not reported | Self reported gum disease in 4.45% HG, 1.31% non-HG, 6 (1.31%) P = 0.0075 | Not reported | Not reported | Not reported | Not reported | Not examined | Self-reported questionnaire relating to “dental cavities” | Significantly higher pre- and post-pregnancy reporting of dental cavities and gum disease (gingivitis/periodontal issues) in HG women | Pre-pregnancy cavities: OR 1.41 (95 % CI 1.07–1.87) | Positive association: HG linked to increased dental cavities and gum disease both before and after pregnancy |
| Beevi,2015 | Hypnosis sessions at weeks 16, 20, 28, 36 | Not reported | Not reported | Not reported | Not reported | Not reported | Not reported | Yes, ptyalism gravidarum | Ruled out through clinical exam and lab tests | Yes, constant spitting caused embarrassment and difficulty at work/socially | Clinical exam to rule out infection; self-reported symptoms | Hyperemesis associated with inability to swallow saliva, worsening ptyalism | Not applicable (single case report) | Positive association between hyperemesis and hypersalivation |
| Suzuki,2013 | None (observational study) | Not reported | Not reported | Not reported | Not reported | Not reported | Not reported | Yes, ptyalism gravidarum cases |  | Required spitting cups, tissue, or cloths throughout pregnancy | Clinical records review (retrospective chart analysis) | Ptyalism gravidarum strongly associated with history of hyperemesis gravidarum (adjusted OR 13.5, p<0.01) | Adjusted OR 13.5 (HG history), OR 5.02 (male infant), OR 4.17 (SGA infant) | Positive association between HG history and ptyalism gravidarum |
| Suzuki_2009 | Case 1: Chewing gum / ice chips only | None | Not reported | Not reported | Not reported | Not reported | Not reported | Primary complaint-excessive salivation requiring spitting cups/tissues | Not reported | Reported bad taste and difficulty swallowing thickened saliva | Clinical observation, patient report of daily saliva volume | Both cases had severe nausea/vomiting (HG) preceding PG; no objective oral health indices measured | Not reported | Positive association-persistent PG follows severe HG |
| Robertson_1999 | None (observational); exposure = vomiting frequency | None-study focused on serum biochemistry | Not reported | Not reported | Not reported | Not reported | Not reported | Not reported | Not reported | Not reported | Fasting serum samples analysed for total amylase and pancreatic amylase | 24 % (7/29) of HG patients had raised total serum amylase (range 90–140 U/L); all had normal pancreatic amylase, implying salivary origin of the increase | Proportion with raised amylase | Positive association: repeated vomiting (HG) → salivary hyperamylasaemia |
| Freeman1994 | Phenothiazine (promethazine) followed by belladonna alkaloid | Not reported | Not reported | Not reported | Not reported | Not reported | Yes, mild xerostomia as side effect of treatment | Yes, sialorrhea up to 1.5–2 L/day | Excoriations in buccal cavity and angles of mouth (Case 1) | Yes, bitter taste, frequent expectoration, excoriation | Clinical observation, patient self-report | Excessive salivation worsened nausea/vomiting; hyperemesis and sialorrhea linked | Not applicable (case reports) | Positive association between hyperemesis and sialorrhea |
| Nozaka1990 | Exposure: maternal conditions during pregnancy (e.g., threatened abortion, severe hyperemesis gravidarum, anaemia, drugs) and childhood illnesses within 1 year | Visual examination, photographic evaluation, classification of enamel hypoplasia | Not specifically reported | Not reported | Not reported | Not reported | Not reported | Not reported | Not directly measured; systemic childhood illnesses considered | Not reported | Clinical visual inspection, photographic evaluation, questionnaire survey | Maternal severe hyperemesis gravidarum during embryogenesis identified as a potential cause of enamel hypoplasia in children | Not explicitly quantified in abstract; associations described qualitatively | Positive association between maternal severe hyperemesis gravidarum and enamel hypoplasia |

|  | **Mechanisms/Explanations Proposed by Authors (if any)** | **Conclusions** | **Limitations** | **Level of Evidence** | **Comments** |
| --- | --- | --- | --- | --- | --- |
| Köşüş,2021 | Gustin (CA-6) supports taste-bud integrity; altered taste sensation might influence nausea/vomiting. | Salivary gustin does not appear to play a role in HG etiopathogenesis; larger studies including zinc and other appetite regulators are warranted. | High drop-out rate (40 %) reduced power. | Level III (prospective case–control) | Evaluated salivary gustin (CA-6) in HG; highlights a non-significant trend but underscores need for larger, multi-variable investigations. |
| Altas,2020 | • Two-way vicious cycle: periodontitis → systemic inflammation → may exacerbate HG; HG-induced vomiting → impaired oral hygiene → worsening periodontal indices. • Hormonal changes alone unlikely to explain rapid attachment loss. | Gingival inflammation is more common in HG; periodontal preventive programs are crucial for pregnant women with HG. | Small single-centre sample (n = 60)  Cross-sectional design → cannot infer causality  Short gestational window (8–10 week)  No adjustment for confounders (nutrition, psychosocial factors)  No microbiological or systemic inflammatory biomarkers measured | Level IV (cross-sectional study) | Study to specifically examine periodontal status in women with HG versus healthy pregnant controls; highlights potential bidirectional HG–periodontal disease relationship but needs larger longitudinal or interventional studies. |
| Bronshtein,  2018 | Hormonal (β-hCG, oestrogen), neuronal, or psychological factors; exact mechanism unknown. | PG is benign for mother/foetus; no adverse perinatal outcomes observed. Hypnosis+acupuncture may help, but evidence limited. | Retrospective design Small sample from a single private clinic No objective quantification of saliva volume No dental or oral health assessments Possible selection or recall bias | Level IV (retrospective cohort) | Focuses on hypersalivation rather than traditional oral pathologies. First study to report PG–HG overlap in Israeli population. Dental researchers could consider evaluating oral soft-tissue effects of chronic hypersalivation in future PG studies. |
| Tian,2017 | Authors suggest chronic vomiting/reflux (GERD) and poor oral hygiene during HG may contribute; no direct oral examinations performed | HG is associated with higher rates of self-reported dental cavities and gum disease, highlighting oral health as an under-recognized comorbidity in HG | Self report bias | Level III-Case-control study | US dataset linking HG to pre-existing and persisting oral health issues; suggests routine dental screening may benefit HG patients |
| Beevi,2015 | Anxiety and fear of vomiting prevent swallowing saliva, worsening ptyalism | Hypnosis successfully eliminated hyperemesis and ptyalism; improved psychological well-being | Single case; long gaps between sessions; cannot establish causality | Low (case report) Level V | First report of hypnosis used for ptyalism gravidarum with hyperemesis |
| Suzuki,2013 | Progesterone and hCG may influence salivary viscosity; possible genetic mechanism linked to male foetal sex | Ptyalism gravidarum may be a distinctive condition leading to adverse perinatal outcomes, especially SGA infants | Small case number (n=22); retrospective design; lack of maternal hCG measurements | Moderate (retrospective cohort study) Level IV | Incidence of ptyalism gravidarum in Japan: ~0.3% |
| Suzuki,2009 | Parasympathetic overstimulation → excessive, water-rich saliva | PG is rare, benign but distressing; no established effective treatment; further physiologic studies needed | Small number of cases | Level V- Case report | Highlights extreme end of the PG spectrum; underscores need for larger cohorts and oral health assessments in HG-PG studies |
| Robertson,1999 | Parotid gland stimulation by chronic vomiting → increased salivary amylase release and glandular hypertrophy | Vomiting (in HG or bulimia) is sufficient to raise salivary amylase; clinicians should recognise that hyperamylasaemia in pregnancy may be salivary, not pancreatic, avoiding unnecessary investigations | Small number of cases | Level IV-observational comparative study | demonstrated salivary hyperamylasemia in hyperemesis gravidarum, highlighting salivary gland response to vomiting as a potential systemic biomarker |
| Freeman1994 | Possible hormonal (HCG, oestrogens, thyroid), neural, and psychological influences; treatment needed to control vomiting before salivation improved | Combined antiemetic and antimuscarinic therapy eliminated both hyperemesis and sialorrhea | Two cases only; limited generalizability; potential medication side effects (xerostomia, constipation) | Low (case reports) Level V | Sialorrhea reported only in Haitian patients within clinic population; possible cultural/genetic factors |
| Nozaka1990 | Enamel hypoplasia often began during matrix formation; maternal conditions (hyperemesis, anaemia, threatened abortion, drugs) and childhood illnesses interact with timing and individual sensitivity | Multiple maternal and childhood conditions contribute to enamel hypoplasia; severe maternal hyperemesis gravidarum is one contributing factor | Observational design; recall bias in questionnaires; no direct measurement of maternal exposures; study in Japanese population only | Moderate (large observational field survey) Level IV | Highlights maternal hyperemesis gravidarum as a risk factor for enamel hypoplasia in offspring |
