# Supplementary material for: Hyperemesis gravidarum and oral health: A scoping review
Source: Int J Gynaecol Obstet. 2026 Mar 31;175(1):48–55. doi: 10.1002/ijgo.70958 (PMC13629587; doi:10.1002/ijgo.70958)
Supplement: Supplementary file 3 [file IJGO-175-48-s002.doc]

Appendix 2: Sample Search Strategy

**Pubmed**

(( "Hyperemesis Gravidarum"[MeSH] OR "Nausea in Pregnancy"[MeSH] OR "Morning Sickness"[MeSH] OR "severe NVP"[tiab] OR "nausea and vomiting of pregnancy"[tiab] OR "morning sickness"[tiab] OR "intractable vomiting"[tiab] OR "pernicious vomiting"[tiab] OR "pregnancy sickness"[tiab] OR "hyperemesis"[tiab] )) AND (( "Oral Health"[MeSH] OR "Dental Caries"[MeSH] OR "Tooth Erosion"[MeSH] OR "Periodontal Diseases"[MeSH] OR "Saliva"[MeSH] OR "Xerostomia"[MeSH] OR "Oral Hygiene"[MeSH] OR "oral health"[tiab] OR dent*[tiab] OR tooth*[tiab] OR gingiv*[tiab] OR perio*[tiab] OR caries[tiab] OR "acid erosion"[tiab] OR saliva[tiab] OR xerostomia[tiab] OR hypersaliva*[tiab] OR "oral hygiene"[tiab] OR "dental care"[tiab] OR "oral diseas*"[tiab] ))

**Scopus**

( ( TITLE-ABS-KEY ( "Hyperemesis Gravidarum" ) ) OR ( TITLE-ABS-KEY ( "Nausea in Pregnancy" ) ) OR ( TITLE-ABS-KEY ( "Morning Sickness" ) ) OR ( TITLE-ABS-KEY ( "severe NVP" ) ) OR ( TITLE-ABS-KEY ( "nausea and vomiting of pregnancy" ) ) OR ( TITLE-ABS-KEY ( "morning sickness" ) ) OR ( TITLE-ABS-KEY ( "intractable vomiting" ) ) OR ( TITLE-ABS-KEY ( "pernicious vomiting" ) ) OR ( TITLE-ABS-KEY ( "pregnancy sickness" ) ) OR ( TITLE-ABS-KEY ( "hyperemesis" ) ) ) AND ( ( TITLE-ABS-KEY ( "Oral Health" ) ) OR ( TITLE-ABS-KEY ( "Dental Caries" ) ) OR ( TITLE-ABS-KEY ( "Tooth Erosion" ) ) OR ( TITLE-ABS-KEY ( "Periodontal Diseases" ) ) OR ( TITLE-ABS-KEY ( "Saliva" ) ) OR ( TITLE-ABS-KEY ( "Xerostomia" ) ) OR ( TITLE-ABS-KEY ( "Oral Hygiene" ) ) OR ( TITLE-ABS-KEY ( "oral health" ) ) OR ( TITLE-ABS-KEY ( dent* ) ) OR ( TITLE-ABS-KEY ( tooth* ) ) OR ( TITLE-ABS-KEY ( gingiv* ) ) OR ( TITLE-ABS-KEY ( perio* ) ) OR ( TITLE-ABS-KEY ( caries ) ) OR ( TITLE-ABS-KEY ( "acid erosion" ) ) OR ( TITLE-ABS-KEY ( saliva ) ) OR ( TITLE-ABS-KEY ( xerostomia ) ) OR ( TITLE-ABS-KEY ( hypersaliva* ) ) OR ( TITLE-ABS-KEY ( "oral hygiene" ) ) OR ( TITLE-ABS-KEY ( "dental care" ) ) OR ( TITLE-ABS-KEY ( "oral diseas*" ) ) )

**Web of Science**

TS=(
 "Hyperemesis Gravidarum" OR
 "Nausea in Pregnancy" OR
 "Morning Sickness" OR
 "severe NVP" OR
 "nausea and vomiting of pregnancy" OR
 "pregnancy sickness" OR
 hyperemesis OR
 "intractable vomiting" OR
 "pernicious vomiting"
 )
 AND
 TS=(
 "oral health" OR
 "dental caries" OR
 "tooth erosion" OR
 "enamel erosion" OR
 "periodontal disease*" OR
 "oral hygiene" OR
 "dental care" OR
 "oral disease*" OR
 saliva* OR
 xerostomia OR
 hypersaliva* OR
 dent* OR
 tooth* OR
 gingiv* OR
 perio* OR
 caries
 )

**OVID Medline**

1.Hyperemesis Gravidarum/
 2. Nausea/
 3. Vomiting/
 4. ("Hyperemesis Gravidarum" or "nausea and vomiting of pregnancy" or "morning sickness" or "pregnancy sickness" or hyperemesis or "severe NVP" or "ptyalism gravidarum" or "vomiting in pregnancy")
5. 1 or 2 or 3 or 4

6. Oral Health/

7. Dental Caries/

8. Tooth Erosion/

9. Periodontal Diseases/

10. Saliva/

11. Xerostomia/

12. Oral Hygiene/

13. ("oral health" or "dental caries" or "tooth erosion" or "enamel erosion" or "periodontal disease*" or gingivitis or "oral hygiene" or "dental care" or "oral disease*" or saliva* or xerostomia or hypersaliva* or ptyalism or dent* or tooth* or gingiv* or perio* or caries).ti,ab,kw.

14. 6 or 7 or 8 or 9 or 10 or 11 or 12 or 13

15. 5 and 14

**Embase**

Hyperemesis Gravidarum / NVP

1. 'hyperemesis gravidarum'

2. 'nausea'

3. 'vomiting'

4. ('hyperemesis gravidarum' or 'nausea and vomiting of pregnancy' or 'morning sickness' or 'pregnancy sickness' or hyperemesis or 'severe NVP' or 'ptyalism gravidarum' or 'vomiting in pregnancy')

5. ((nausea or vomiting) adj3 pregnan*)

6. 1 or 2 or 3 or 4 or 5

Oral Health

7. 'oral health'

8. 'dental caries'

9. 'tooth erosion'

10. 'periodontal disease'

11. 'saliva'

12. 'xerostomia'

13. 'oral hygiene'

14. ('oral health' or 'dental caries' or 'tooth erosion' or 'enamel erosion' or 'periodontal disease*' or gingivitis or 'oral hygiene' or 'dental care' or 'oral disease*' or saliva* or xerostomia or hypersaliva* or ptyalism or dent* or tooth* or gingiv* or perio* or caries)

15. 7 or 8 or 9 or 10 or 11 or 12 or 13 or 14

16. 6 and 15

17. 'pregnancy'

18. pregnan*

19. 17 or 18

20. 16 and 19

**CINAHL Ultimate**

S1

("Hyperemesis Gravidarum+")

OR

TX ("Hyperemesis Gravidarum" OR "nausea and vomiting of pregnancy" OR "morning sickness" OR "pregnancy sickness" OR hyperemesis OR "severe NVP" OR "ptyalism gravidarum" OR "gestational vomiting" OR "vomiting in pregnancy" OR "pregnancy-related nausea")

OR

TX ((nausea OR vomiting) N3 pregnan*)

S2

("Oral Health+")

OR ("Dental Caries+")

OR ("Tooth Erosion+")

OR ("Periodontal Diseases+")

OR ("Saliva+")

OR ("Xerostomia+")

OR ("Oral Hygiene+")

OR

TX ("oral health" OR dental OR "oral manifestation*" OR "oral complication*" OR "tooth erosion" OR "dental erosion" OR "enamel erosion" OR perimylolysis OR "tooth wear" OR "periodontal disease*" OR gingivitis OR saliva* OR hypersaliva* OR ptyalism OR xerostomia OR "oral hygiene" OR "oral disease*" OR caries OR dent* OR tooth* OR gingiv* OR perio*)

S3

S1 AND S2

**Cochrane library**

‘Hyperemesis gravidarum’ AND ‘oral diseas*’
